# Clinical Research Skills Assessment: An Investigation into the Determinants of Effective Research

**DOI:** 10.7759/cureus.1870

**Published:** 2017-11-22

**Authors:** Mohamad Al-Tannir, Isamme AlFayyad, Amani Abu-Shaheen, Ahmed Al-Badr, Fatimah Al Mousawi

**Affiliations:** 1 Research Center, King Fahad Medical City

**Keywords:** educational needs, health care providers, knowledge, research, saudi arabia

## Abstract

Background

Developing individual research skills and enhancing the institutional research culture leads to quality research capabilities and research excellence at the national level. We aim to assess the educational needs of healthcare providers regarding research skills at King Fahad Medical City (KFMC), Riyadh, Saudi Arabia.

Methods

From February 2016 to October 2016, we conducted a cross-sectional study using a self-administered questionnaire of the healthcare providers at KFMC. The questionnaire targeted staff who have not been involved in research (n=353; “category-1”), staff who received Institutional Review Board (IRB) approval (n=94; “category-2”), and staff who have completed and published their research (n=53; “category-3”). A descriptive analysis was used to measure the frequency, and the chi-square test was used to test significance when comparing categorical data.

Results

The final analysis contained 500 questionnaires. The number of women was higher than that of men in “category-2 “and “category-3” (53.2%, 62.3%), respectively. Approximately 62.4% of “category-1” participants reported good, poor, and very poor knowledge of epidemiology. Participants in “category-1” and “category-2” stated poor and very poor levels when writing a manuscript (43%, 23.4%), respectively. Only 37% of participants in “category-3” showed very good to excellent research skills. However, there was a significant correlation between the mean scores of research skill and research stage (p-value < 0.001).

Conclusion

The results showed a significant variation in research skills needs among research stage categories; therefore, meeting the educational needs of healthcare providers aimed at effective research shall be constructed based on their stage of research.

## Introduction

Recently, there was an increasing focus to boost health research and evidence-based practice to achieve the national health goals, primarily in developing countries. A robust health research system is significant for an efficient health system [1]. Moreover, clinical research influences the prevention, diagnosis, and treatment of diseases [2].

The development of research capacity is primarily based on the development of individual research skills and the enhancement of institutional research culture, which ultimately leads to quality research capacity and research excellence at the national level [3]. An appraisal of the research skills of developing researchers has shown that some significant research skills are essential for competence in conducting research. These research skills include, but are not limited to, information seeking, communicating (e.g., protocols submission and manuscript writing skills), methodological skills, and data analysis (e.g., correct data analysis and statistics) [4].

Vigorous and energetic research personnel are imperative to Saudi’s future prosperity in the research arena. Our researchers and research scientists constitute crucial talent that needs to be acknowledged and supported. King Fahad Medical City (KFMC), as a reference medical care institution, provides specialized patient care by being active in training, education, and research. These activities are mutually connected and inter-reliant to build an effective research environment, support current research investigators, and establish a platform for future research scholars. In addition, continuous professional academic achievements are associated with research skills training and experience in the early periods of an individual’s professional life [5-6].

Numerous studies have been conducted to assess the research skills and knowledge of nurses in the healthcare system. A previous study surveyed more than 400 nurses in six different health care settings within the UK and showed that approximately 93% of the nurses were not satisfied with their research knowledge and skills [7]. Moreover, Pravikoff et al. and Koehn et al. revealed a deficiency of skills among nurses to search, appraise, and produce research literature, a difficulty in understanding research papers, and an absence of knowledge/partial knowledge of research [8-9].

Research experience is vital to a healthcare provider’s evidence-based practice, as it conveys skills such as literature review and data collection and analysis, and evidence of critical appraisal [10-11]. In a national mailed study in Canada, Leahy et al. found that family physicians varied in their ideas regarding the significance of research education during residency [12].

The literature recommends that professional learning and development is continued when the education is allied with the practice, whereby individual motivation energizes the educational work [13]. Therefore, we propose that the first step in promoting the research culture and practice is to assess the educational needs of health care providers and plan a continuous process of learning.

Few studies have examined the importance of evolving a researcher’s skills. Kardash designed a tool to capture the evolving research skills of undergraduates involved in research activities [14]. In addition, Powers and Enright created an instrument to measure graduate student research skills [15].

Given the need to develop an operational plan and a distinctive, effective research program that recognizes the vital role research personnel play in supporting quality discoveries and advances, a survey was conducted to assess the educational needs of healthcare providers regarding research knowledge and skills at KFMC.

## Materials and methods

Study design

Following Institutional Review Board (IRB) approval, a cross-sectional study was conducted at KFMC using a self-administered questionnaire from February 2016 to October 2016.

Study participants and sample size

The questionnaire targeted three categories among healthcare providers, according to their stage of research activity, including 1) staff who have never been involved in research activities “category-1”, 2) staff who have received IRB approval but have not finished their projects “category-2”, and 3) staff who have completed and published their research “category-3.” The sample size was calculated according to research categories. The sample size of “category-1” was based on the total number of healthcare providers at KFMC (353 participants). The “category-2” sample size was calculated based on the number of approved studies from the IRB office, which was found to be 94 participants. The sample size of “category-3” was calculated based on the number of completed and published studies through the KFMC research center (53 participants). Participants were approached by a trained research assistant and asked to sign informed consent forms and to complete a self-administered questionnaire. Verbal consent was obtained from each participant before filling out the survey.

Data collection methods, instruments, and measurements

The questionnaire was developed after an in-depth review of the literature. Before commencing work, a pilot study was conducted to assess the reliability of the questionnaire. Cronbach’s alpha resulted in more than 0.9.

The questionnaire was constructed in three parts. Part one was composed of participant characteristics, including age, gender, department (Women Specialized Hospital (WSH); Children Hospital (CH); Rehabilitation Hospital (RH); Main Hospital, Comprehensive Cancer Center (CCC); King Salman Heart Center (KSHC); National Neuroscience Institute (NNI); Obesity, Metabolic and Endocrine Center (OMEC); Out Patient Department (OPD), and other ancillary services), profession, and occupation. Part two looked at the level and origin of the academic degree, total years of experience, and involvement at KFMC. The third part addressed the stages of research activity, the number of research participants, and the assessment of skills needed for effective research. The assessment of the participant’s skills was assessed using a five-point Likert scale, ranging from “Excellent – 1”, “Very Good – 2”, “Good – 3”, “Poor – 4”, and “Very Poor – 5.” The research skills questions evaluated the following areas: 1) basic concepts of research and epidemiology, 2) appraisal of medical literature, 3) effective literature search, 4) basics of biostatistics, 5) writing a grant proposal, 6) writing a scientific manuscript, 7) submitting a manuscript to a journal, 8) addressing journal reviewers comments, 9) research monitoring, 10) presentation skills (e.g., oral vs. poster), 11) communication skills, 12) data records and management, and 13) knowledge of good clinical practice guidelines (GCP). A mean research skills score of ≤ 2 (very good and excellent) was considered a satisfactory research skill level; higher scores were considered unsatisfactory.

Statistical analysis

Data analysis was conducted using SPSS 22.0 software (SPSS Inc., Chicago, IL, USA). The arithmetic mean was used as a summary to assess the level of research skills. The chi-square test was used to determine the strength of the association between the categories of research stages and research skills. For all statistical analysis, p ≤ 0.05 was considered statistically significant.

## Results

A total of 500 questionnaires were collected and entered into the final data analysis. Table 1 shows the participant’s demographics and characteristics. A total of 353 (70.6%) participants have not been involved in any research activity: “category-1.” The majority of participants in “category-2” and “category-3” (90.1% and 81.1%, respectively) have participated in fewer than five studies. Among all categories, more than two-thirds of the participants were younger than 40 years of age. Women were more active in research than men in “category-2” and “category-3” (53.2% and 62.3%, respectively).

**Table 1:** Characteristics of participants

		Stage of research			
		Never involved in research n (%)	Received IRB approval n (%)	Completed and published research n (%)	p-values
Stage of research activity		353 (70.6)	94 (18.8)	53 (10.6)	
How many research studies have you participated in?	1-5	0	73 (90.1)	43 (81.1)	
	5-10	0	5 (6.2)	2 (3.8)	
	>10	0	3 (3.7)	8 (15.1)	
	Total	353 (100)	81 (100)	53 (100)	
Age	≤30	144 (45.9)	27 (30.7)	18 (36.7)	0.065
	31 - 40	100 (31.8)	43 (48.9)	22 (44.9)	
	41 - 50	62 (19.7)	15 (17.0)	7 (14.3)	
	>50	8 (2.5)	3 (3.4)	2 (4.1)	
	Total	314 (100)	88 (100)	49 (100)	
Gender	Male	48 (13.8)	44 (46.8)	20 (37.7)	<0.001
	Female	301 (86.2)	50 (53.2)	33 (62.3)	
	Total	349 (100)	94 (100)	53 (100)	
Department	WSH	69 (20.1)	7 (7.9)	10 (23.3)	0.002
	CH	16 (4.7)	1 (1.1)	3 (7.0)	
	RH	61 (17.7)	6 (6.7)	5 (11.6)	
	Main hospital	68 (19.8)	23 (25.8)	6 (14.0)	
	CCC	31 (9.0)	10 (11.2)	4 (9.3)	
	KSHC	18 (5.2)	14 (15.7)	3 (7.0)	
	NNI	17 (4.9)	11 (12.4)	6 (14.0)	
	OMEC	17 (4.9)	4 (4.5)	2 (4.7)	
	OPD	0 (0.0)	0 (0.0)	0 (0.0)	
	Others	47 (13.7)	13 (14.6)	4 (9.3)	
	Total	344 (100)	89 (100)	43 (100)	
B.Sc. degree origin	Middle East	81 (27.6)	53 (61.6)	19 (38.8)	<0.001
	South/Far East	212 (72.1)	24 (27.9)	23 (46.9)	
	West	1 (0.3)	9 (10.5)	7 (14.3)	
	Total	294 (100)	86 (100)	49 (100)	
M.Sc. degree origin	Middle East	26 (70.3)	19 (47.5)	7 (30.4)	0.002
	South/Far East	9 (24.3)	5 (12.5)	6 (26.1)	
	West	2 (5.4)	16 (40.0)	10 (43.5)	
	Total	37 (100)	40 (100)	23 (100)	
Ph.D. degree origin	Middle East	5 (41.7)	5 (41.7)	0 (0.0)	0.009
	South/Far East	7 (58.3)	2 (16.7)	1 (20.0)	
	West	0 (0.0)	5 (41.7)	4 (80.0)	
	Total	12(100)	12 (100)	5 (100)	
Profession	Physician	45 (13.0)	38 (43.2)	15 (33.3)	<0.001
	Nurse	284 (81.8)	34 (38.6)	24 (53.3)	
	Pharmacist	14 (4.0)	12 (13.6)	3 (6.7)	
	Laboratory Specialist	4 (1.2)	4 (4.5)	3 (6.7)	
	Total	347 (100)	88 (100)	45 (100)	
Occupation	Consultant	2 (1.0)	14 (20.6)	8 (20.5)	<0.001
	Assistant Consultant	9 (4.5)	12 (17.6)	3 (7.7)	
	Fellow	17 (8.4)	5 (7.4)	4 (10.3)	
	Resident	25 (12.4)	6 (8.8)	1 (2.6)	
	Head nurse	143 (70.8)	22 (32.4)	17 (43.6)	
	Senior Pharmacist	3 (1.5)	7 (10.3)	3 (7.7)	
	Senior lab Specialist	3 (1.5)	2 (2.9)	3 (7.7)	
	Total	202 (100)	68 (100)	39 (100)	
Total years of experience	<5	92 (26.4)	24 (25.5)	15 (28.8)	0.739
	5 - 10	129 (37.1)	29 (30.9)	18 (34.6)	
	≥10	127 (36.5)	41 (43.6)	19 (36.5)	
	Total	348	94	52	
Years of experience at KFMC	<5	199 (58.9)	42 (45.2)	37 (71.2)	0.032
	5 - 10	90 (26.6)	35 (37.6)	11 (21.2)	
	≥10	49 (14.5)	16 (17.2)	4 (7.7)	
	Total	338 (100)	9 (100)	52 (100)	
Abbreviations: CCC, Comprehensive Cancer Center; CH, Children Hospital; IRB, Institutional Review Board; KFMC, King Fahad Medical City; KSHC, King Salman Heart Center; NNI, National Neuroscience Institute; OMEC, Obesity, Metabolic and Endocrine Center; OPD, Outpatient Department; RH, Rehabilitation Hospital; WSH, Women Specialized Hospital.					

Our results revealed that among bachelor degree holders, more staff from the Middle East (61.6%) were involved in “category-2” and more staff from the South/Far East (46.9%) were involved in “category-3.” Among staff with Ph.D. degrees, Middle Easterners and Westerners displayed similar involvement in “category-2”; however, staff with Western certificates were more involved in “category-3.” Participating physicians were mainly grouped in “category-2” and “category-3”; meanwhile, the majority of nurses (81.8%) have never been involved in a research activity. Staff with more than 10 years total experience were grouped in “category-2” and “category-3” (43.6% and 36.5%, respectively); however, staff with under five years of experience at KFMC were placed in “category-2” (45.2%) and “category-3” (71.1%). A statistically significant association was found between participant gender, department, origin of academic degree, profession, occupation, and years of experience at KFMC (Table 1). 

Table 2 shows participants’ research skill levels. Approximately 62.4% of “category-1” participants reported good, poor, or very poor (combined) knowledge of the basic concepts of epidemiology. However, more than two-thirds of participants in “category-2” and “category-3” reported excellent or very good (combined) levels of research skills. A total of 49.5% of participants in “category-1” reported that they have an excellent or very good (combined) level of medical literature review. However, participants in “category-2” and “category-3” have a better understanding of the medical literature (76.6% and 67.9%, respectively) compared with “category-1.” “Category-1” participants were average in effective literature searches (50.6% scored good, poor, or very poor). Furthermore, 30.2% of participants in “category-2” and 32.1% in “category-3” reported good, poor, or very poor (combined) research skills in effective literature searching. Our results determined that statistical analysis skills were higher in “category-2” and “category-3” participants.

**Table 2:** Distribution of participants' responses for their level of research skills

		Stage of research		
		Category-1 n(%)	Category-2 n(%)	Category-3 n(%)
Basic concepts of research and epidemiology	Excellent	19 (5.4)	25 (26.6)	11 (20.8)
	Very good	113 (32.2)	37 (39.4)	23 (43.4)
	Good	212 (60.4)	31 (33.0)	19 (35.8)
	Poor	6 (1.7)	1 (1.1)	0 (0.0)
	Very poor	1 (0.3)	0 (0.0)	0 (0.0)
	Total	351 (100)	94 (100)	53 (100)
Appraisal of medical literature	Excellent	24 (6.9)	28 (29.8)	12 (22.6)
	Very good	149 (42.6)	44 (46.8)	24 (45.3)
	Good	171 (48.9)	19 (20.2)	17 (32.1)
	Poor	5 (1.4)	3 (3.2)	0 (0.0)
	Very poor	1 (0.3)	0 (0.0)	0 (0.0)
	Total	350 (100)	94 (100)	53 (100)
Effective literature search	Excellent	41 (11.6)	30 (32.3)	11 (20.8)
	Very good	133 (37.8)	34 (36.6)	25 (47.2)
	Good	166 (47.2)	28 (30.1)	16 (30.2)
	Poor	11 (3.1)	1 (1.1)	1 (1.9)
	Very poor	1 (0.3)	0 (0.0)	0 (0.0)
	Total	352 (100)	93 (100)	53 (100)
Basics of biostatistics	Excellent	17 (5.0)	8 (9.2)	8 (15.1)
	Very good	103 (30.3)	45 (51.7)	21 (39.6)
	Good	198 (58.2)	31 (35.6)	22 (41.5)
	Poor	21 (6.2)	3 (3.4)	2 (3.8)
	Very poor	1 (0.3)	0 (0.0)	0 (0.0)
	Total	340 (100)	87 (100)	53 (100)
Writing a grant proposal	Excellent	2 (0.6)	2 (2.1)	9 (17.0)
	Very good	28 (8.0)	26 (27.7)	14 (26.4)
	Good	165 (47.3)	46 (48.9)	21 (39.6)
	Poor	145 (41.5)	17 (18.1)	8 (15.1)
	Very poor	9 (2.6)	3 (3.2)	1 (1.9)
	Total	349 (100)	94 (100)	53 (100)
Writing a scientific manuscript	Excellent	1 (0.3)	3 (3.2)	5 (9.4)
	Very good	26 (7.4)	29 (30.9)	17 (32.1)
	Good	155 (44.4)	40 (42.6)	24 (45.3)
	Poor	157 (45.0)	18 (19.1)	6 (11.3)
	Very poor	10 (2.9)	4 (4.3)	1 (1.9)
	Total	349 (100)	94 (100)	53 (100)
Submitting a manuscript to a journal	Excellent	2 (0.6)	3 (3.2)	9 (17.0)
	Very good	20 (5.8)	18 (19.4)	10 (18.9)
	Good	146 (42.2)	34 (36.6)	24 (45.3)
	Poor	165 (47.7)	32 (34.4)	8 (15.1)
	Very poor	13 (3.8)	6 (6.5)	2 (3.8)
	Total	346 (100)	93(100)	53 (100)
Addressing journals' reviewer comments	Excellent	5 (1.4)	4 (4.5)	9 (17.0)
	Very good	25 (7.2)	20 (22.5)	11 (20.8)
	Good	172 (49.6)	41 (46.1)	24 (45.3)
	Poor	136 (39.2)	23 (25.8)	8 (15.1)
	Very poor	9 (2.6)	1 (1.1)	1 (1.9)
	Total	347 (100)	89 (100)	53 (100)
Research monitoring	Excellent	4 (1.1)	6 (6.6)	11 (20.8)
	Very good	44 (12.6)	29 (31.9)	18 (34.0)
	Good	187 (53.7)	43 (47.3)	18 (34.0)
	Poor	111 (31.9)	12 (13.2)	6 (11.3)
	Very poor	2 (0.6)	1 (1.1)	0 (0.0)
	Total	348 (100)	91 (100)	53 (100)
Presentation skills (oral vs. posters)	Excellent	18 (5.1)	21 (22.3)	14 (26.4)
	Very good	77 (21.9)	41 (43.6)	20 (37.7)
	Good	212 (60.4)	26 (27.7)	17 (32.1)
	Poor	41 (11.7)	6 (6.4)	1 (1.9)
	Very poor	3 (0.9)	0 (0.0)	1 (1.9)
	Total	351 (100)	94(100)	53(100)
Communication skills	Excellent	31 (8.8)	33 (35.1)	15 (28.3)
	Very good	123 (34.9)	41 (43.6)	24 (45.3)
	Good	187 (53.1)	18 (19.1)	14 (26.4)
	Poor	9 (2.6)	2 (2.1)	0 (0.0)
	Very poor	2 (0.6)	0 (0.0)	0 (0.0)
	Total	352 (100)	94 (100)	53 (100)
Data records and management	Excellent	18 (5.1)	26 (28.6)	13 (24.5)
	Very good	90 (25.6)	35 (38.5)	21 (39.6)
	Good	207 (59.0)	24 (26.4)	16 (30.2)
	Poor	33 (9.4)	6 (6.6)	3 (5.7)
	Very poor	3 (0.9)	0 (0.0)	0 (0.0)
	Total	351 (100)	91 (100)	53 (100)
Knowledge of good clinical practice guidelines	Excellent	5 (1.5)	8 (8.9)	12 (24.0)
	Very good	44 (13.0)	39 (43.3)	12 (24.0)
	Good	206 (60.8)	33 (36.7)	20 (40.0)
	Poor	74 (21.8)	9 (10.0)	4 (8.0)
	Very poor	10 (2.9)	1 (1.1)	2 (4.0)
	Total	339 (100)	90 (100)	50 (100)

More than half (combined) of the participants among each of the three categories reported good or below in grant proposal writing skills. When participants were asked about their skill of writing a scientific manuscript, participants in “category-1” and “category-2” reported poor or very poor (43% and 23.4%, respectively). Nevertheless, only 13.2% of participants in “category-3” revealed poor or very poor levels in scientific manuscript writing. When asked about their skill at addressing a journal reviewer’s comments, 17% of the participants in “category-3” indicated poor or very poor skills, and only 37% of participants in “category-3” showed very good or excellent skills.

Approximately 11.1% of participants in “category-1”, 12% in “category-2”, and 24.1% in “category-3” reported a poor or very poor understanding of GCP guidelines. In addition, Table 2 shows that there was a significant difference in research skill among the categories of participants.

Table 3 shows that participants in “category-1” have an unsatisfactory level of all research skills (mean scale score ≥ 2). Moreover, participants in “category-2” demonstrate satisfactory research skills in their appraisal of medical literature, effective literature searching, and communication skills. However, participants in “category-3,” who have completed and published studies, display a satisfactory level of communication skills.

**Table 3:** Correlation of mean research skill scores with research stage categories *Satisfactory research skills mean score (≤2 out of 5) Abbreviation: GCP, good clinical practices.

	Category-1	Category-2	Category-3	p-value
	(Mean ± SD)			
Basic concepts of epidemiology	2.59±0.634	2.09±0.799	2.15±0.744	<0.001
Appraisal of medical literature	2.46±0.657	1.97±0.796*	2.09±0.741	<0.001
Effective literature search	2.43±0.748	2.00±0.779*	2.13±0.820	<0.001
Basics of biostatistics	2.66±0.682	2.33±0.693	2.34±0.783	<0.001
Writing a grant proposal	3.38±0.694	2.93±0.820	2.58±1.008	<0.001
Writing a scientific manuscript	3.43±0.685	2.90±0.893	2.64±0.879	<0.001
Submitting a manuscript to a journal	3.48±0.690	3.22±0.942	2.70±1.049	<0.001
Addressing journals' reviewer comments	3.34 ±0.714	2.97±0.845	2.64±1.002	<0.001
Research monitoring	3.18±0.695	2.70±0.823	2.36±0.942	<0.001
Presentation skills (oral vs. posters)	2.81±0.736	2.18±0.855	2.15±0.907	<0.001
Communication skills	2.51±0.716	1.88±0.788*	1.98±0.747*	<0.001
Data records and management	2.75±0.728	2.11±0.900	2.17±0.871	<0.001
Knowledge of GCP	3.12±0716	2.51±0.838	2.44±1.072	<0.001

## Discussion

In this cross-sectional study, we aimed to assess the educational needs of healthcare providers regarding research skills. Our results showed that there is a significant variation in research knowledge and skills among participants based on experience in the three categories of research stages. 

The pattern of research productivity varies among researchers of different age and gender [16-18]. The results of our study at KFMC revealed that participants in “category-2” and “category-3” were under age 40, and that research productivity decreased with age, though the decline was statistically insignificant (p = 0.065). Moreover, we found that women were more involved in research activities than men. However, a study conducted to assess the impact of gender on research productivity showed that the academic women’s research is equal to men's productivity [19]. Therefore, we assume that age and gender are influencing factors in research productivity.

We noticed a negative trend between years of experience at KFMC and research activity among participants in “category-2” and “category-3,” where research activity declined as years of experience increased.

Research requires quality skills in scientific manuscript writing that leads to publication in an indexed journal. The most important research skill required is an understanding of the basic concepts of epidemiology. Adequate knowledge of epidemiological methods is essential for conducting a study and analyzing data derived from clinical studies [20]. The results of the present study revealed that approximately one-third of the participants in “category-2” and “category-3” do not have a high quality (i.e., very good and excellent) level of the basic concepts of epidemiology, which certainly will undermine the quality of the research produced. Healthcare providers are challenged with an overabundance of scientific papers that focus on various clinical questions and subjects, evaluating clinical remedies, and discovering the predictive value of several factors in therapeutic outcomes [20]. This practice requires a considerable level of skill by the reader to research and appraise the study design, research methodology, data analysis, and interpretation of findings of pertinent research to reach conclusions. We examined the skills of medical literature search and appraisal and found that it was at an unsatisfactory level in 50% of the participants in “category-1.” Meanwhile, about two-thirds of the participants in “category-2” and “category-3” have a higher research skill level in medical literature search and appraisal, which could be explained by their exposure to research and their research experience. 

In healthcare disciplines, understanding statistical analysis has essential effects in modifying clinical practice, as it has a great implication on evidence-based diagnostic and treatment modalities [20]. However, among all participants in each of the three categories, there was a lack of basic biostatistics, which is supported by previous studies that have shown that physicians and pharmacists have limited and poor knowledge of biostatistics and face difficulties in comprehending the value of epidemiological implication [21-24].

While knowledge and skills are a necessity to conduct research, research funding is crucial to cover research expenses and procedures. Our study showed that most participants in “category-1” lacked grant proposal writing skills. Participants in “category-2” and “category-3” showed poor or very poor grant writing skills. Principally, writing a good research proposal does not mean it will get funded, however, a well-written research proposal can support studies, if approved [25].

The definitive purpose of writing a manuscript is to publish study results and improve knowledge. Indexed journals require a high-quality scientific manuscript for publication. However, writing a manuscript can be exciting and frustrating, which can be discouraging for a novice researcher [26]. The skill level for writing a scientific manuscript between all categories of participants was unsatisfactory, which will lower their chances for publication.

Publishing scientific results is an essential part of a researcher’s career. Nevertheless, writing is not every researcher’s preferred activity; publishing a scientific article can be boring and time-consuming [27]. Although the peer review process is unlikely to alter the core nature of a submitted manuscript, in several circumstances, the authors may be required to “add analysis or results, clarify thoughts or parameters, revise the statistical testing methods, increase the number of subjects, or lengthen the time of clinical follow-up in response to the reviewer’s requests” [28]. Participants in “category-1” showed poor or very poor levels when submitting a manuscript to a journal in terms of fulfilling the journal requirements.

GCP guidelines are recognized internationally as ethical and scientific quality standards. GCP aims to manage and organize the designing, conducting, recording, and reporting of clinical trials involving human subjects. GCP guidelines provide public assurance that the correct safety and well-being of trial subjects are protected and that the data resulting from the trial are complete, accurate, and unbiased [29-30]. Our results revealed a remarkable proportion of poor or very poor knowledge of GCP guidelines among all categories. Although Al-Nomay reported a satisfactory level of GCP knowledge among healthcare professionals in Saudi Arabia, a poor compliance rate was found among study participants [29-30].

Building research capacity is a necessity for public healthcare systems endeavoring to provide outstanding quality care, and evidence-based practice necessitates research to be part of the healthcare system. Education in the above-delineated research skills will shift healthcare providers from untrained or novice researchers to quality researchers and from education to innovation. Our structured set of research skills is common to the success of researchers; it is comprehensive and addresses all research stages. Research skill workshops and education addressing the set of research skills required for success could potentially advance the knowledge of medical professionals regarding research [5].

Research capacity building is the principal operational objective of the KFMC research enterprise. Developing quality researchers and a supportive environment are the main themes of capacity building. Research center services ultimately focusing on providing support in research protocol planning, grant proposal writing, and statistical consultative services for data management create governing and informative policies. In addition, research centers and healthcare environments need to conduct in-service educational workshops related to all aspects of research processes, including infrastructures in the form of research laboratories, equipment, software, and conference support.

The main limitation of our study was that we did not explore the reasons behind poor research knowledge and skills among healthcare providers. Our study aimed to assess research educational needs among healthcare providers from different educational backgrounds. Therefore, it is recommended to conduct future studies exploring potential reasons. Moreover, our study was conducted in a single medical care hospital; hence, conducting a multicenter study is recommended to have a more generalizable result.

## Conclusions

The results obtained from this self-administered questionnaire determine that the educational needs for effective research skills are obvious. The identified educational needs are the cornerstone for constructing a needs-based educational program. Our results showed that there is a significant variation in research skills among researchers; therefore, meeting the educational needs of healthcare providers in order to perform effective research should be constructed based on the appropriate stage of research.
